# TNFα-Related Chondrocyte Inflammation Models: A Systematic Review

**DOI:** 10.3390/ijms251910805

**Published:** 2024-10-08

**Authors:** Su Wang, Sarah Kurth, Christof Burger, Dieter C. Wirtz, Frank A. Schildberg, Robert Ossendorff

**Affiliations:** Department of Orthopedics and Trauma Surgery, University Hospital Bonn, 53127 Bonn, Germany

**Keywords:** TNFα, inflammation model, cartilage, chondrocyte, cytokine

## Abstract

Tumor necrosis factor alpha (TNFα), as a key pro-inflammatory cytokine, plays a central role in joint diseases. In recent years, numerous models of TNFα-induced cartilage inflammation have been developed. However, due to the significant differences between these models and the lack of consensus in their construction, it becomes difficult to compare the results of different studies. Therefore, we summarized and compared these models based on important parameters for model construction, such as cell source, cytokine concentration, stimulation time, mechanical stimulation, and more. We attempted to analyze the advantages and disadvantages of each model and provide a compilation of the analytical methods used in previous studies. Currently, TNFα chondrocyte inflammation models can be categorized into four main types: monolayer-based, construct-based, explant-based TNFα chondrocyte inflammation models, and miscellaneous TNFα chondrocyte inflammation models. The most commonly used models were the monolayer-based TNFα chondrocyte inflammation models (42.86% of cases), with 10 ng/mL TNFα being the most frequently used concentration. The most frequently used chondrocyte cell passage is passage 1 (50%). Human tissues were most frequently used in experiments (51.43%). Only five articles included models with mechanical stimulations. We observed variations in design conditions between different models. This systematic review provides the essential experimental characteristics of the available chondrocyte inflammation models with TNFα, and it provides a platform for better comparison between existing and new studies in this field. It is essential to perform further experiments to standardize each model and to find the most appropriate experimental parameters.

## 1. Introduction

Articular cartilage consists of chondrocytes surrounded by an extracellular matrix (ECM). This tissue has an optimized composition to withstand mechanical forces. However, articular cartilage is aneural and avascular, and chondrocytes are nourished by the diffusion of synovial fluid, resulting in a low healing potential of the tissue after injury. Osteoarthritis (OA) is a major disease characterized by cartilage loss, subchondral bone changes, osteophyte formation, and persistent inflammation. The inflammatory response affects all of the joint structures, including cartilage, bone, and synovial tissue. Tumor necrosis factor alpha (TNFα) is a potent pro-inflammatory cytokine that has been implicated in many diseases, and it plays a critical role in the immune system during inflammation [1]. TNFα is mainly produced by activated macrophages, T cells, and natural killer cells, but it can also be secreted by chondrocytes. It exists in two forms: a soluble form of 17 kDa and a transmembrane form of 26 kDa [2]. The precursor form, the transmembrane form (tm-TNFα), is converted to the soluble form (sTNFα) to enable biological activities through the action of the TNFα-converting enzyme (TACE) [3]. TNFα interacts with its two distinct receptors, designated as TNFR1 and TNFR2 (TNFR1, also known as TNFRSF1A, CD120a, and p55; TNFR2, also known as TNFRSF1B, CD120b, and p75), which initiate signaling pathways leading to various cellular responses, including cell survival, differentiation, and proliferation and migration [4,5,6,7]. TNFα can inhibit cartilage matrix synthesis and increase its degradation by promoting the release of matrix metalloproteinases (MMPs), and it inhibits chondrogenesis through the nuclear factor-κB (NF-κB) pathway by preventing the synthesis of SOX9, a transcription factor required for the chondrocyte phenotype [8,9,10,11,12,13]. Given that TNFα-neutralizing therapies are effective in the treatment of autoimmune and chronic inflammatory diseases, the importance of TNFα in inflammation has been highlighted [14].

The aim of this review was to provide an overall summary of experimental approaches that have investigated the pro-inflammatory effect of TNFα on cartilage/chondrocyte tissue in vitro. This will allow for better referencing when developing new models or tactics and may also improve the translation of research from bench to bedside.

## 2. Results

### 2.1. Description of Studies

**Figure 1:**
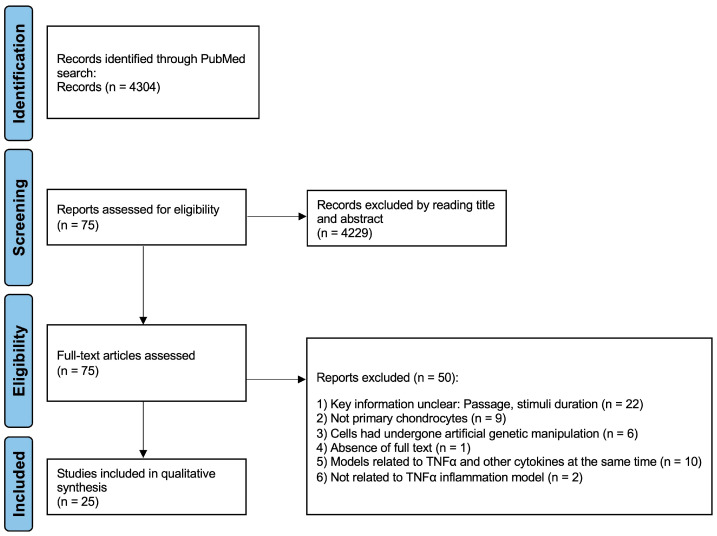
Identification and selection of studies via databases and registers.

A total of 4304 reports were identified and 75 were selected for further review after reading the full text (Figure 1). In total, 25 studies met the inclusion criteria and were included in this systemic review and 50 studies were excluded. Twenty-two studies were excluded because some key information on the TNFα inflammation model was missing (chondrocyte passage and/or stimulus duration) [15,16,17,18,19,20,21,22,23,24,25,26,27,28,29,30,31,32,33,34,35,36]. Nine studies did not use primary chondrocytes [37,38,39,40,41,42,43,44,45]. One study was not accessible as a full text. Ten studies used TNFα together with other cytokines, such as IL-1β, to create an inflammation model and were therefore excluded. Two studies were not related to TNFα inflammation models. Six articles were excluded due to genetic modification. More specific model details are provided in Table 1, Table 2, Table 3 and Table 4.

### 2.2. Classification of TNFα Chondrocyte Inflammation Models

**Figure 2:**
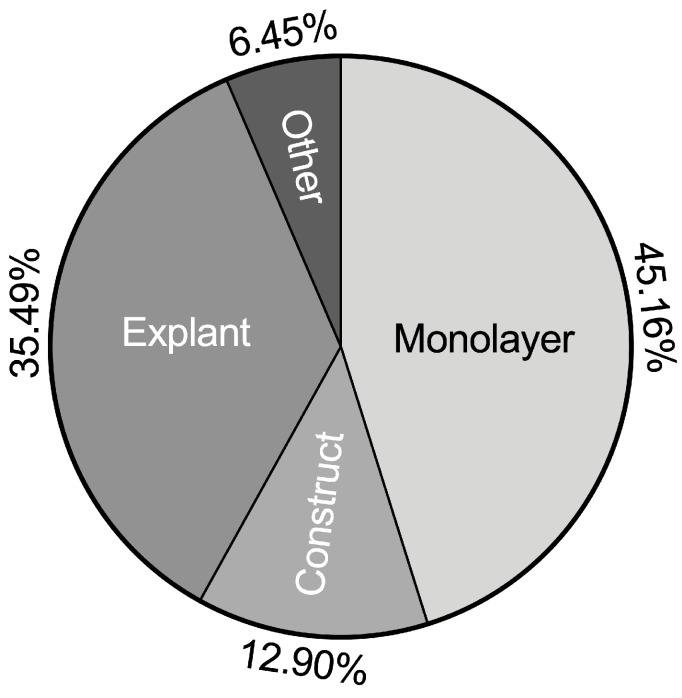
Summary of TNFα chondrocyte inflammation models.

TNFα-related chondrocyte/cartilage inflammation models can be divided into four broad categories according to the method of model construction (Figure 2): (1) monolayer-based TNFα chondrocyte inflammation models (45.16%), (2) explant-based TNFα chondrocyte inflammation models (35.49%), (3) construct-based TNFα chondrocyte inflammation models (12.90%), and (4) other inflammation models (6.45%).

### 2.3. Concentrations and Stimulation Durations of TNFα

**Figure 3:**
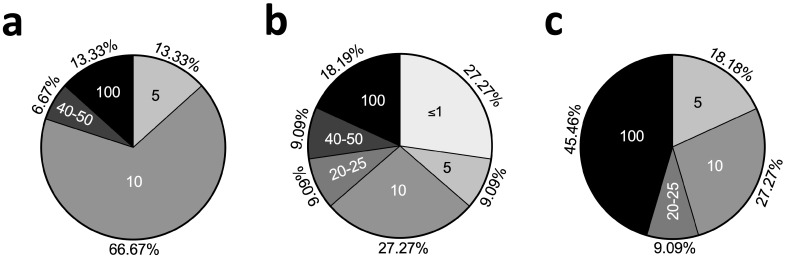
Concentration of TNFα in ng/mL used in different models (**a**): monolayer-based TNFα chondrocyte inflammation models, (**b**): construct-based TNFα chondrocyte inflammation models, and (**c**) explant-based TNFα chondrocyte inflammation models.

The choice of cytokine concentration would depend on the choice of model. The most commonly used TNFα concentration in monolayer-based, construct-based TNFα chondrocyte inflammation models was 10 ng/mL, but in explant-based TNFα chondrocyte inflammation models, the concentration was 100 ng/mL (Figure 3).

**Figure 4:**
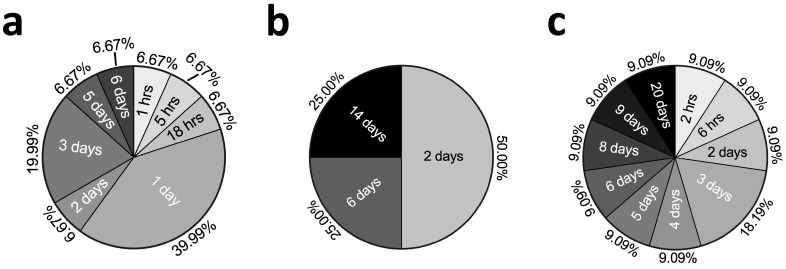
Summary of TNFα stimulation durations in hours and/or days (**a**): monolayer-based models, (**b**): construct-based models, and (**c**): explant-based models.

The duration of TNFα stimulation also depended on the type of model. In general, the stimulation time for monolayer-based models was shorter than the others (less than one week in all reported studies). In more than half of these studies, the stimulation time did not exceed 48 h, with 24 h being the most commonly used stimulation time (Figure 4).

For construct-based TNFα chondrocyte inflammation models, the shortest stimulation time was 48 h, which was also the most common stimulation time, and the longest stimulation time was 14 days. For explant-based TNFα chondrocyte inflammation models, the shortest time was 2 h [57], and the longest was 20 days, with the largest range of stimulation times between the studies (Figure 4).

### 2.4. Passages Used in TNFα Chondrocyte Inflammation Models

**Figure 5:**
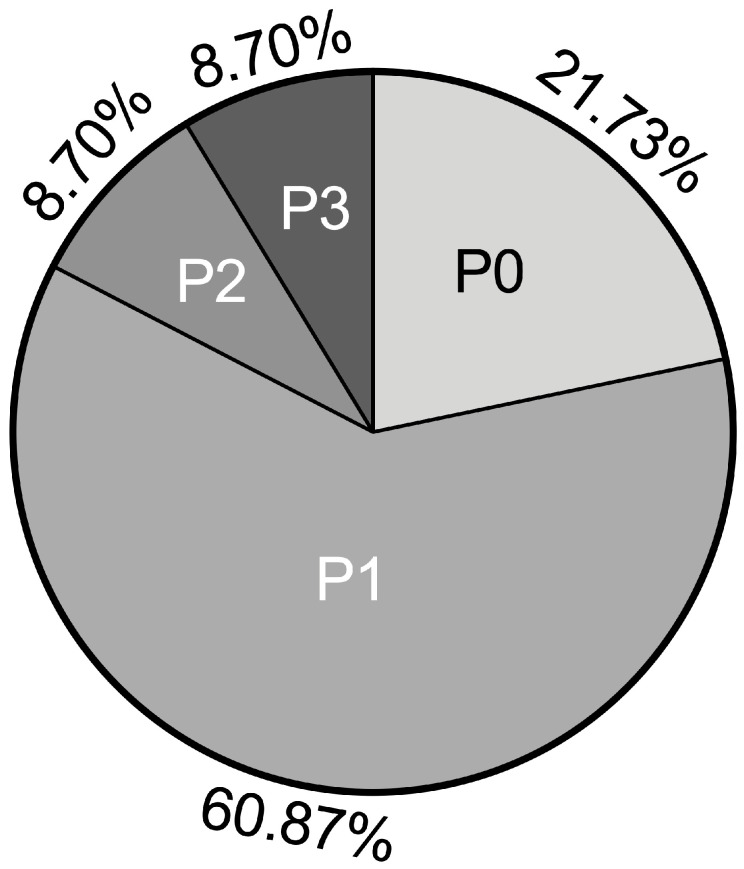
Passages’ (P0–P3) distribution of chondrocytes TNFα inflammation models.

The chondrocyte passages used in the reported studies varied from passage 0 to 3. Passage 1 (60.87%) and passage 0 (21.73%) were the most commonly used (Figure 5).

### 2.5. Species Distribution

**Figure 6:**
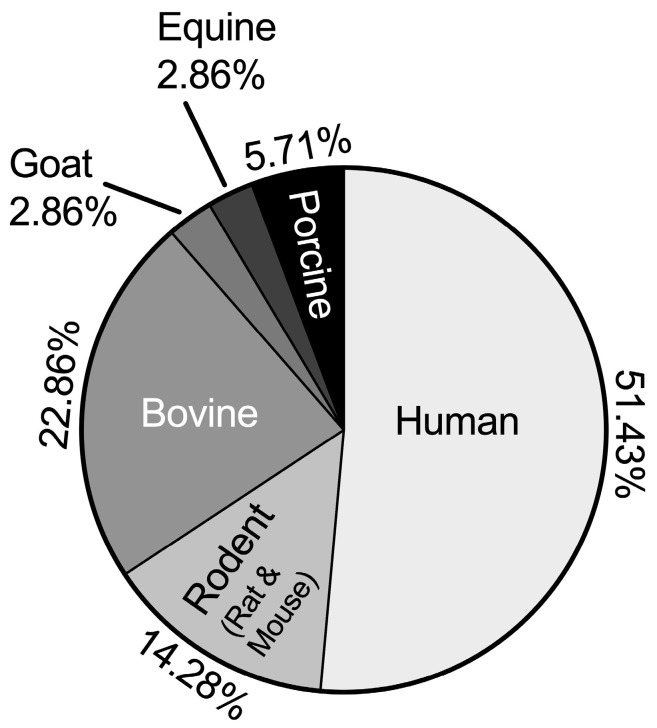
Species distribution of TNFα chondrocyte inflammation models.

The species used in TNFα chondrocyte inflammation models in all of the reviewed literature included human (51.43%), rodent (14.28%), goat (2.86%), bovine (22.86%), equine (2.86%), and porcine (5.71%) (Figure 6).

### 2.6. Mechanical Stimuli

Five studies dealt with mechanical stimulation [61,62,66,67,68]. We summarized the different mechanical stimuli on the basis of the type of load, the type and magnitude of the load, the frequency and duration of the load, the experimental setting, and the device used to deliver the mechanical stimuli. The parameters used varied between the studies and models. In summary, the experiments primarily utilized either a construct-based or an explant-based TNFα chondrocyte inflammation model to investigate cartilage/chondrocyte responses under various conditions and loading scenarios. See Supplementary Table S1 for further details.

### 2.7. Common Methods of Evaluating Experimental Results

We summarized the target genes identified by PCR from the included literature [46,50,51,53,58,61,64,65,66,70]. Monolayer-based models used a wider variety of genes (16 in total). The target genes selected in the three models overlapped somewhat, with collagen type 2, aggrecan, matrix metalloproteinases (MMP-3, MMP-13), a disintegrin and metalloproteinase with thrombospondin motifs 5 (ADAMTS5), and TNFα appearing in almost all of the models (Supplementary Table S2).

For tissue staining techniques, four histological staining methods were used to access cartilage degradation [47,58,60,61,68]. These were toluidine blue staining, safranin O/fast green staining, alcin blue staining, and picrosirus red staining. Toluidine blue was the most commonly used method (Supplementary Table S3).

The target proteins in the immunohistochemical analysis varied from the structural proteins collagen type 1, collagen type 2, and aggrecan to cartilage matrix degrading enzymes, including MMP-3 and MMP-13, and signaling pathway proteins [47,50,51,58,59,61]. Among these proteins, collagen type 2 was the most abundant. More specific results are shown in Supplementary Table S4.

We also summarized the signaling pathways frequently addressed in the literature [50,56,57,69,71,72,73,74,75,76,77,78]. These studies encompassed several pathways, including the NF-κb, MAPK, Jak-STAT, PI3K-Akt, and HIF pathways. The NF-κb and MAPK pathways were the most frequently investigated pathways (Supplementary Table S5).

## 3. Discussion

In this review, we systematically analyzed TNFα-related models of inflammation in chondrocytes and cartilage tissue. These models were classified into four main categories: monolayer-based, construct-based, explant-based TNFα chondrocyte inflammation models, and miscellaneous models. Among these, monolayer-based TNFα chondrocyte inflammation models (45.16%) were the most prevalent. The human species (51.43%) was most frequently identified in the included studies. Furthermore, the concentration of added TNFα and the duration of cytokine stimulation were model-dependent. The most commonly used concentrations for the monolayer, construct, and explant-based TNFα chondrocyte inflammation models were 10 ng/mL, 10 ng/mL, and 100 ng/mL, respectively (66.67%, 27.27%, and 45.46%). In addition, the most commonly used stimulation durations for monolayer-based and construct-based TNFα chondrocyte inflammation models were 24 h, 48 h, and 3 days, respectively (39.99%, 50.00%, and 18.19%). The chondrocytes used to construct the models were typically derived from passages 0–3, with passage 1 accounting for the majority (60.87%). We also summarized the mechanical parameters used to construct TNFα chondrocyte inflammation models, PCR gene targets, cartilage histology staining methods, immunohistochemistry targets, and pathways involved in these studies.

### 3.1. TNFα Chondrocyte Inflammation—Differences in Available Models

Different models have different strengths and are suitable for studying different aspects of the disease. Monolayer-based TNFα chondrocyte inflammation models have a precise regulation of chondrocyte culture composition and concentration, as well as oxygen pressure. However, monolayer cultures are not physiological and affect the functional phenotype of the cells and the resulting tissue [79,80]. Chondrocytes lose their cartilage phenotype and express a fibroblast phenotype during monolayer cell culture. Roman-Blas et al. [70] and Shakibaei et al. [69] maintained the chondrocyte phenotype by suspension culture and by combining 2D and 3D cultures. The disadvantage of the inability of this model to receive mechanical stimuli can also be overcome by combining this model with a 3D model and a joint-specific bioreactor [81].

Construct-based TNFα inflammation models could provide a 3D environment for chondrocytes. Cells are seeded into the constructs, which promotes chondrogenic differentiation. Mechanical stimulation can be applied to the constructs to better mimic the living environment of cartilage in the body. The scaffold materials for this model are the most critical. The material should be biocompatible, not cause severe inflammatory reactions, and allow adequate diffusion of nutrients to the cells [82]. The materials typically used for constructs are agarose, alginate, and hyaluronic acid, and, for scaffolds, polyethylene glycol, acrylamide hydrogels, or other porous materials [83,84]. The most common types of cells seeded are primary chondrocytes or differentiated chondrocytes derived from mesenchymal stromal cells. Mohanraj et al. [60] used an agarose construct model and showed that chondrocytes derived from bone marrow stem cells (MSCs) were more easily influenced by the inflammatory environment compared to native chondrocytes.

Cartilage explant models mimic the cytokine response in a relatively natural environment, allowing the degradation of the extracellular matrix to be monitored [79]. And it is easier to accept mechanical stimulation that can induce chondrogenesis [85,86]. The disadvantage is that the volume of cartilage from small animals such as the mouse is limited, and the cartilage structure is different between small animals and humans. The cartilage structure, biochemical composition, and functional properties of large animals are closer to those of humans. These models include porcine or equine species, but these experiments are more time consuming, costly, and usually involve ethical issues [87]. Animal samples can be obtained from animal models with standardized induced disease simulation or from completely healthy animals. Human cartilage tissue is usually obtained from healthy patients as organ donors after an accident or from patients with late-stage OA who undergo an arthroplasty procedure. These differences in phenotype are important to consider when comparing studies. In addition, organ donors often do not provide completely healthy cartilage, which is more likely to be expected from juvenile donors. Again, the age of the donor is important. It is therefore difficult to obtain completely healthy or relatively healthy cartilage, and such human cartilage tissue is not suitable for modeling the early stages of inflammatory diseases. There is also the problem that the quality of the sample depends on the site from which it is taken. It has been suggested that cartilage tissue from the knee joint loses more GAGs than cartilage tissue from the ankle joint when subjected to the same mechanical stimuli [68]. And cells at the edge of the cartilage explant are more likely to die than those in the center, skewing the observations at these sites [88].

The lack of harmonization in the construction of TNFα inflammation models makes it difficult to compare the results of different studies. Based on the results of this review, the commonly used TNFα concentration (100 ng/mL) for explant-based models of these studies is higher than that (10 ng/mL) for monolayer and construct-based models. While the most commonly used concentration remains the same for monolayer and construct-based models, the duration of TNFα stimulation (48 h) for construct-based models is longer than that (24 h) for monolayer-based models.

In this review, five studies reported the type of recombinant TNFα used [46,47,59,66,68]. It is unclear whether the use of different species of recombinant TNFα in homologous models results in differences. The need to use recombinant TNFα that is homologous to the experimental cells/cartilage remains to be clarified. The lack of uniformity in model construction makes it difficult to compare the results between the studies.

### 3.2. High Diversity of Outcome Assessments in Chondrocyte Inflammation Models with TNFα

In general, the primary goals of TNFα chondrocyte inflammation models are either to study the pathology of cartilage inflammatory diseases, to investigate the underlying mechanisms, or to test or improve the efficacy of treatments. Techniques such as histological assessment, biomarker measurement, and molecular biology analysis have proven useful in achieving these goals. For some models, mechanical assessments can play an important role.

The common genes associated with TNFα chondrocyte inflammation models are summarized in Supplementary Table S2. Genes can be categorized into several classes: those related to cartilage matrix components and their synthesis, such as aggrecan, PRG4, collagen type 2, collagen type 1, collagen type X, and SOX9; genes associated with extracellular matrix remodeling and turnover, such as MMPs, ADAMTS5, and their tissue inhibitors of metalloproteinases (TIMPs); and genes associated with the inflammatory response, such as iNOS and cyclooxygenase-2 (COX-2), interleukin (IL)-6, and IL-8. The selection of gene targets varied between the models, but there is no doubt that the matrix-related genes collagen type 2, aggrecan, MMP-3, MMP-13, and ADAMTS 5 are critical genes in the study of cartilage inflammation and degeneration.

Proteoglycans and collagen are important components of healthy joint cartilage [89]. In addition to HE staining, histological staining techniques include picrosirius red staining to visualize the collagen network in the extracellular matrix, and alcian blue and toluidine blue staining for sulfated glycosaminoglycans (sGAG), a critical component of proteoglycans. A combination of safranin-O and fast green staining is also used to highlight the presence of glycosaminoglycans. These staining methods provide a qualitative visualization of the spatial distribution of collagen and proteoglycans within the tissue. Different models show variations in the choice of staining methods.

Immunohistochemistry (IHC) can also be used to understand the distribution and content of different collagen fibers using antibodies against different collagen fibers (e.g., collagen type 1 and collagen type 2). Of the three models, collagen type 2 was selected as the target protein in all cases. When performing IHC, a 3D model is usually chosen. This is because there is more matrix to stain. When chondrocytes are cultured in a monolayer, there is little matrix secreted. Interestingly, however, Chen et al. [56] performed IHC staining of collagen type 2 by culturing cells on coverslips to identify chondrocytes.

In addition to histology and IHC, biochemical assays such as the dimethylmethylene blue (DMMB) method can assess the degradation of sulfated glycosaminoglycans in the cartilage matrix. These methods evaluate changes in the cartilage matrix composition from both a quantitative and qualitative perspective.

In 3D models, mechanical assessments are essential to understand the mechanical response of cartilage tissue or constructs to various stimuli. Compression tests and the determination of Young’s modulus are commonly used to assess mechanical properties [83,90,91,92]. In addition, there are other ways to measure mechanical properties that are related to specific experimental models. In the study by Bevill et al. [66], creep consolidation and the cyclic strain of cartilage were tested to assess the mechanical properties of cartilage tissue after mechanical stimulation. Djouad et al. [58] used a push-out test to determine the interface strength between the chondrocyte-loaded construct and natural cartilage.

### 3.3. Mechanical Stimulation as Key for the Simulation of Physiologic Conditions

In the physiological context, articular cartilage is routinely subjected to a variety of mechanical forces, including compression, shear, hydrostatic pressure, and tensile stress; it is dynamic, intermittent, and multi-axial. An adequate mechanical force is required to maintain extracellular homeostasis for chondrocytes. However, it is important to avoid overloading, as excessive mechanical stress can trigger apoptosis or inflammatory cascade within the tissue [79,93,94,95].

Regarding the duration of mechanical loading, it is common for mechanical injury simulations to involve a single application at the beginning of the experiment, followed by continued experiments with the injured specimens. In contrast, the duration of regular mechanical stimuli may vary depending on the specific research objectives.

Dynamic, intermittent, and multi-axial mechanical loading is often used to mimic the mechanical stresses experienced by articular cartilage during daily activities. The choice of frequency in mechanical loading experiments should be based on the research objectives and the physiological conditions you are trying to mimic. Lower frequencies (e.g., 0.01–1 Hz) are closer to the frequency of activities such as walking, rather than higher applied frequencies (e.g., 1–10 Hz). However, researchers should also consider the variations between the different models and research objectives. As different joint cartilage tissues experience varying levels of mechanical pressure under physiological conditions, the level of mechanical stress should be determined based on the experimental objectives and sample types. For example, during activities such as walking and standing, the knee joint typically experiences pressures in the range of 2 to 12 MPa, while the hip joint encounters 1 to 4 MPa. Shoulder joints typically experience 0.5 to 2 MPa. There may be a threshold for enhancing cartilage repair effects, but this threshold varies between the different models [61,66,96,97,98].

### 3.4. Limitations

This systematic review focused on the inflammation model with an external stimulation by the recombinant protein TNFα. There are other inflammation models available, which were not included in this review. In these models, only the stimulus induced by TNFα is taken into account, but not when TNFα is produced in vitro on the basis of the stimulus (e.g., by stimulating chondrocytes with MSU crystals or CPPD). This criticism is part of the fact that the translational significance of using chondrocytes to study cartilage inflammation is not yet clear.

## 4. Materials and Methods

### 4.1. Exclusion and Inclusion Criteria and Study Selection

This systematic review was conducted according to the Preferred Reporting Items for Systematic Reviews and Meta-Analyses (PRISMA) guidelines [99]. A comprehensive search of the electronic medical database PubMed was performed by two independent authors (SK and SW) from 1 August 1986 to 20 July 2023 using the search terms “TNF alpha” OR “TNFα” AND “chondrocyte” OR “cartilage”. Only English titles were included.

Studies included in the systematic review were those that investigated the pro-inflammatory effect of TNFα on primary chondrocytes, engineered chondrocyte constructs, or cartilage explants. A primary screening of titles and abstracts was conducted by including studies of any level of evidence published in peer-reviewed journals reporting results in English. After screening, all the included literature was read in full for further screening against the exclusion criteria. Exclusion criteria included (1) a lack of information on chondrocyte passage, species, stimulus duration, and the dosage of cytokines, or other stimuli. (2) Articles that did not use primary chondrocytes, such as those employing chondrocyte cell lines or chondrocytes derived from mesenchymal stem cells (MSCs), or fibroblast cells, were also excluded. This exclusion was justified for better comparability. (3) Articles using cytokines other than TNFα to induce inflammation were also excluded. (4) Full-text articles that were not accessible were also excluded.

Two authors independently performed the study selection and any discrepancies in the selection process were resolved by discussion. A senior investigator (RO) was consulted to review the selection process.

### 4.2. Data Collection Process

All data were extracted from the article text, tables, and figures. Two investigators independently reviewed each article. Discrepancies between the two reviewers were resolved through discussion and consensus. The final results were then reviewed by the senior investigator (RO). Data were extracted from each included report, including information on (1) the species of chondrocyte or cartilage, (2) the passage number of chondrocytes, (3) concentration and type of TNFα used, (4) the duration of each stimulus, (5) the type of intervention (including the type of TNFα, concentration, duration, and mechanical stimuli), (6) the type of inflammation model used, (7) the main results reported, and (8) the PCR primers used in the experiment.

## 5. Conclusions

Currently, there is a wide variety of TNFα chondrocyte inflammation models. In this review, models are categorized into four main groups: monolayer-based, construct-based, explant-based TNFα chondrocyte inflammation models, and other types of models. Different models need to be selected depending on the objective of the research. Meanwhile, specific factors such as species selection, TNFα concentration, and stimulation period, whether mechanical stimulation is used, vary between the models. The lack of standardization in these TNFα chondrocyte inflammation models makes comparisons between the models difficult. More systematic studies are needed to standardize the specific models. This systematic review provides an overview of the existing studies and is a reference for a better comparison of TNFα-induced inflammatory models.

## Figures and Tables

**Table 1:** Two-dimensional models: Monolayer-based TNFα chondrocyte inflammatory models.

Species	Authors	Origin	Age (Mean)	Concentration (ng/mL)	Passage	Duration	Main Findings
Human	Kim et al. [46]	Knee OA	69.5 y	10	1	24 h	Sulforaphane (SFN) hindered the synthesis of prostaglandin and nitric oxide in human articular chondrocytes, thereby preventing the degradation of cartilage matrix.
Human	Malemud et al. [47]	OA Adult knee Juvenile knee	NA NA 12 y	10	1	1 h	TNFα could increase apoptosis in normal human chondrocytes, OA chondrocytes and human juvenile chondrocyte pellet cultures, but not in chondrocyte pellet cultures initiated from MSCs.
Human	López-Armada et al. [48]	Healthy knee	59 y	10	1	6 days	TNFα and IL1β impacted the mitochondrial function of human articular chondrocytes, and the inhibition of complex I may be a factor in the cartilage breakdown caused by these cytokines.
Human	López-Armada et al. [49]	Hip OA	NA	10	1	7 days	TNFα and IL-1β exerted distinct influences on the apoptotic pathway in human chondrocytes, with this variation being contingent upon the levels of PGE2 and caspase-8.
Human	Boileau et al. [50]	Healthy knee Knee OA	52 y 76 y	5	1	72 h	Activation of PAR-2 in osteoarthritic cartilage influenced disease pathways, making PAR-2 antagonists promising for OA treatment.
Human	Tardif et al. [51]	Healthy cartilage OA	66 y 71 y	5	1	24 h	Chordin was regulated differently in normal and osteoarthritic human chondrocytes.
Human	Caramés et al. [52]	Healthy cartilage	NA	10	1	24 h	TNFα and IL-1β regulated apoptosis differently in human chondrocytes.
Human	Yik et al. [53]	Knee OA	44–80 y	10	0	5 h	CDK-9 activity was required for the primary inflammatory response in chondrocytes, CDK-9 inhibition provides protection of cartilage against catabolism effects of proinflammatory cytokines.
Human	Ding et al. [54]	Healthy ankle	45 y	100	2	24 h	DAMPs induced chondrolysis with cytokines or fibronectin fragment, but lost their effects when acting alone.
Human	Terkeltaub et al. [55]	Healthy knee OA knee	NA NA	10	1	18 h	Maintaining AMPK activation protected the cartilage matrix from deterioration caused by inflammation.
Rat	Chen et al. [56]	Wild type	4 w	10	3	72 h	Electroacupuncture serum inhibits TNFα mediated chondrocyte inflammation via the Ras-Raf-MEK1/2-ERK1/2 signaling pathway.
Mouse	Cao et al. [57]	Wild type Transgenic	4 w 4 w	10	1	1 h	TNFα stimulated mitochondrial superoxide flash activity by 2-fold in vitro and 5-fold in situ. Mitochondria are a significant source of cellular oxidants.
Bovine	Ding et al. [54]	Healthy stifle joint	NA	100	1	24 h	DAMPs induced chondrolysis with cytokines or fibronectin fragment, but lost their effects when acting alone.
Bovine	Djouad et al. [58]	Carpometacarpal joint (healthy)	6 m	10	1	72 h	Proinflammation cytokines had devastating effects on the cartilage constructs and those effects could be inhibited by the blockade of ERK signaling pathway.
Goat	Chen et al. [59]	Healthy knee	NA	40	1	24 h	Proinflammatory cytokine could change the mechanical properties of chondrocyte in vitro.

**Table 2:** Three-dimensional models: Construct-based TNFα chondrocyte inflammatory models.

Species	Authors	Origin	Age (Mean)	Model	Concentration (ng/mL)	Passage	Duration	Main Findings
Bovine	Mohanraj et al. [60]	Healthy knee	2–6 m	Agarose construct + TNFα	1, 5, 10	0	6 days	MSC-derived constructs were more easily influenced by the inflammatory environment compared to chondrocyte-derived constructs.
Bovine	Ossendorff et al. [61]	Healthy fetlock joint	5–7 m	PolyurethanePU scaffolds + TNFα	20	3	14 days	TNFα had negative effects on chondrogenesis under simulated ACI conditions. Dynamic load and adalimumab inhibited those effects.
Bovine	Tilwani et al. [62]	Healthy cartilage	<18 m	Agarose construct + TNFα	0.1, 10, 100	0	48 h	TNF enhanced NO, PGE2, and MMP activity at 5% oxygen tension, and dynamic compression might counteract this impact.
Human	Morris et al. [63]	Healthy knee and ankle	65 y	alginate beads + TNFα	1, 10, 50, 100	0	48 h	TIMP3 may serve a chondroprotective role and it may be necessary to bind to ECM for its full function.

**Table 3:** Three-dimensional models: Explant-based TNFα chondrocyte inflammatory models.

Species	Authors	Origin	Age	Concentration (ng/mL)	Duration	Main Findings
Human	Boileau et al. [50]	Healthy knee OA knee	52 y 76 y	5	2 days	PAR-2 activation had been linked to catabolic and inflammatory processes associated with OA development.
Human	Kim et al. [46]	OA knee	69.5 y	10	9 days	Sulphoraphane (SFN) inhibited multiple catabolic mechanisms in cartilage.
Human	Tardif et al. [51]	Healthy cartilage OA Cartilage	66 71	5	3 days	Chordin was regulated differently in normal and osteoarthritic human chondrocytes.
Mouse	Cao et al. [57]	Hip and Knee wild type/transgenic	4 w	10	2 h	TNFα stimulated mitochondrial superoxide flash activity by 2-fold in vitro and 5-fold in situ. Mitochondria are a significant source of cellular oxidants.
Mouse	Terkeltaub et al. [55]	Healthy Femoral Head, wild-type	2 m	10	3 days	Maintaining AMPK activation protected the cartilage matrix from deterioration caused by inflammation.
Horse	Little et al. [64]	Healthy fetlock joint	2–12 y	100	4 days	Regional difference in response to catabolic cytokines was unlikely to be responsible for the initiation of focal cartilage degeneration in osteoarthritis.
Bovine/Porcine	Little et al. [65]	Healthy fetlock joints	2 w 3–6 m	100	4–20 days (Bovine, Porcine)	In vitro, aggrecan produced from normal and OA cartilage in response to TNFα is cleaved by aggrecanase rather than MMPs.
Porcine	Bevill et al. [66]	Healthy knee	6–8 m	100	6 h	The core and peripheral areas of tibial cartilage have distinct gene expression responses to mechanical strain and TNFα.
Bovine	Stevens et al. [67]	Healthy knee	2–3 w	100	5 days	Overload compression damaged the cartilage matrix and disrupted the cell membrane. IL-1 and TNFα stimulate chondrocytes to release proteins linked with innate immune and stress responses, which may aid in host defense against infections and protect cells from stress-induced damage.
Bovine Human	Sui et al. [68]	Healthy knee Healty ankle	1–2 w 26–61 y	25 (Bovine) 100 (Human)	6 days (Bovine) 8 days (Human)	Mechanical stimulation may enhance the catabolic effects of proinflammatory cytokine. In addition, IL-6/sIL-6R works in tandem with TNFα to cause cartilage deterioration.

**Table 4:** Other TNFα chondrocyte inflammatory models.

Species	Authors	Model	Origin	Age	Concentration (ng/mL)	Passage	Duration	Main Findings
Human	Shakibaei et al. [69]	3D to 2D: chondrocytes migrated from alginate bead construct to monolayer culture + TNFα	Hip joint (femoral neck fracture)	NA	10	2	24 h	Curcumin reduced TNF-induced COX-2 and MMP-9 expression in chondrocytes.
Human	Roman-Blas et al. [70]	2D: Suspension culture + TNFα	OA knee	NA	10	0	2 h	IL-1β or TNFα suppressed Smad3/4 DNA-binding activity. IL-1β or TNFα suppression of TGF-β signaling pathways may no related to Smad7.
Human	Malemud et al. [47]	3D: Chondrocytes pellet + recombinant human TNFα	OA knee adult knee juvenile knee	NA NA 12 y	10, 20, 50	1	48 h	TNFα could increase apoptosis in normal human chondrocytes, OA chondrocytes and human juvenile chondrocyte pellet cultures, but not in chondrocyte pellet cultures initiated from MSCs.
Bovine	Djouad et al. [58]	3D: Agarose construct + cartilage ring	Healthy fetlock joint	6 m	1, 10	1	28 days	Proinflammation cytokines had devastating effects on the cartilage constructs and those effects could be inhibited by the blockade of ERK signaling pathway.

## Data Availability

The original contributions presented in this study are included in the article/Supplementary Material; further inquiries can be directed to the corresponding author.

## Supplementary Materials

The following supporting information can be downloaded at: https://www.mdpi.com/article/10.3390/ijms251910805/s1.
