# Supplementary material for: TNFα-Related Chondrocyte Inflammation Models: A Systematic Review
Source: Int J Mol Sci. 2024 Oct 8;25(19):10805. doi: 10.3390/ijms251910805 (PMC11476358; doi:10.3390/ijms251910805)
Supplement: Supplementary file 1 [file ijms-25-10805-s001.zip › ijms-3169231-supplementary.pdf]

Supplementary Table S1. Mechanical Stimulation related studies

Authors	Concentration	Model	Loading type	Loading mode	Loading magnitude	Loading rate	Frequency	Loading duration and resting period	Duration of experiment	Culture condition	Mechanical testing system
Ossendorff et al. [1]	20 ng/ml TNFα	Construct-based TNFα inflammation model	Dynamic, intermittent Compression +oscillating shear	Multiaxial	A preload of 10% strain + 10%-20% strain	/	1 Hz	1 hr of loading+ 8 hrs of resting +1 hr of loading	2 weeks (compression applied every two days)	37°C, 5% CO_2, and 85% humidity	A custom-fabricated incubator-housed load bioreactor
Tilwani et al. [2]	10 ng/ml TNFα	Construct-based TNFα inflammation model	dynamic intermittent compression	Uniaxial	15% strain	/	1 Hz	10mins of loading+5hrs 50mins resting	6 hrs or 48 hrs	5 or 21 % oxygen tension	An ex vivo bioreactor (Bose ElectroForce, Gillingham, UK)
Bevill et al. [3]	100 ng/ml recombinant porcine TNFα	Explant-based TNFα inflammation model	Unconfined dynamic compression	Uniaxial	100 kPa	/	0.6 Hz	6hrs of loading	48 hrs	37 °C and 5% $C0_2$	A custom-fabricated incubator-housed loading apparatus
Stevens et al. [4]	100ng/ml TNFα	Explant-based TNFα chondrocyte inflammation model	Unconfined compression	Uniaxial	50% strain	1 mm/sec	/	/	5days	/	A custom-fabricated incubator-housed loading apparatus
Sui et al. [5]	25ng/ml TNFα for bovine sample, 100ng/ml TNFα for human sample.	Explant-based TNFα chondrocyte inflammation model	Unconfined compression	Uniaxial	50% strain for bovine samples. 60% strain for human samples.	1 mm/sec	/	/	6days for bovine samples, 8days for human samples.	/	A custom-designed, incubator-housed apparatus

Supplementary Table S2. Gene expression markers used in RT-PCR

	Monolayer-based models	Construct-based models	Explant-based models	Other TNFα-based models
Col1		Ossendorff et al.[1]		
Col2	Djouad et al. [6] Yik et al. [7]	Ossendorff et al. [1]	Bevill et al. [3]	Roman-Blas et al. [8]
Col10		Ossendorff et al. [1]		
COMP	Yik et al. [7]	Ossendorff et al. [1]		
Aggrecan	Djouad et al. [6] Yik et al. [7]	Ossendorff et al. [1]	Bevill et al. [3]	Roman-Blas et al. [8]
PRG4		Ossendorff et al. [1]		
MMP1	Djouad et al. [6] Yik et al. [7]		Bevill et al. [3]	
MMP3	Djouad et al. [6] Yik et al. [7]	Ossendorff et al. [1]	Bevill et al. [3] Little et al. [9] Little et al. [10]	
MMP9	Yik et al. [7]			
MMP13	Djouad et al. [6] Yik et al. [7]	Ossendorff et al. [1] Tilwani et al. [2]	Bevill et al. [3] Little et al.[9] Little et al.[10]	
ADAMTS4	Djouad et al. [6] Yik et al. [7]		Bevill et al. [3] Little et al.[9]	
ADAMTS5	Djouad et al. [6] Yik et al. [7]	Tilwani et al. [2]	Little et al.[10]	
TNFα	Djouad et al. [6]	Ossendorff et al. [1]	Bevill et al. [3]	
IL1b	Djouad et al. [6]	Ossendorff et al. [1]		
COX2	Kim et al. [11]			
mPEGS	Kim et al. [11]			
iNOS	Kim et al. [11] Yik et al. [7]		Little et al.[9]	
TIMP1			Bevill et al. [3]	
TIMP2			Bevill et al. [3] Little et al.[9]	
TIMP3		Morris et al. [12]	Little et al. [9]	
PAR2	Boileau et al. [13]		Boileau et al. [13]	
Chordin	Tardif et al. [14]			
SOX9				Roman-Blas et al. [8]
Smad7				Roman-Blas et al. [8]

(Col1: Collagen 1. Col2: Collagen 2. Col10: Collagen 10. COMP: Cartilage Oligomeric Matrix Protein. AGN: Aggrecan. PRG4: Proteinglycan 4. MMP: Matrix Metalloproteinase. ADAMTS4,5: A Disintegrin And Metalloproteinase with Thrombospondin motif 4, 5. COX-2: Cyclooxygenase-2. mPEGS: Microsomal Prostaglandin E Synthase. iNOS: inducible Nitric Oxide Synthase. TIMP1,2,3: Tissue inhibitor of Metalloproteinase 1,2,3. PAR2: Protease-Activated Receptor 2. SOX9: SRY-Box Transcription Factor 9. Smad7: Mothers Against Decapentaplegic Homolog 7.)

Supplementary Table S3. Common Histological staining methods for accessing cartilage degradation

	Construct-based models	Explant-based models	Other TNFα-based models
Toluidine Blue	Ossendorff et al. [1]	Sui et al. [5]	Malemud et al. [15]
Safranin O/ Fast green	Ossendorff et al. [1]		
Alcin blue	Mohanraj et al. [16]		Djouad et al. [6]
Picrosirus red			Djouad et al. [6]

Supplementary Table S4. Immunohistochemistry staining targets

IHC	Monoalyer-based models	Construct-based models	Explant-based models	Other TNFα-based models
Col1		Ossendorff et al. [1]		
Col2	Chen et al. [17]	Ossendorff et al. [1]	Boileau et al. [13]	Djouad et al. [6] Malemud et al. [15]
ColX				Malemud et al. [15]
AGN				Djouad et al. [6]
MMP-1			Boileau et al. [13]	
MMP-13			Boileau et al. [13]	
PAR2			Boileau et al. [13]	
COX2			Boileau et al. [13]	
STAT3				Malemud et al. [15]
Caspase3		Ossendorff et al. [1]		
Chordin			Tardif et al. [14]	

(Col1: Collagen 1. Col2: Collagen 2. ColX: Collagen X. AGN: Aggrecan. MMP1, 13: Matrix Metalloproteinase1, 13. COX2: Cyclooxygenase 2. PAR2: Protease-Activated Receptor 2. STAT3: Signal Transducer and Activator of Transcription 3.)

Supplementary Table S5. Signaling pathways involved in the included studies

Signaling pathways	Key components	Explanations of key components
NF-κB Pathway [13,18–20]	NF-κB is a transcription factor family that regulates immunological response, inflammation, cell survival, and the expression of many genes involved in these processes.	
	IκBα (Inhibitor of κBα)	IκBα exerts an inhibitory effect on NF-κB when it is not phosphorylated, and the activation of NF-κB involves a critical step is the phosphorylation of IκBα.
	p-IκBα (Phosphorylated IκBα)	
	p65	a subunit of the NF-κB complex. Phosphorylated form of p65 indicates pathway activation.
	p-p65 (Phosphorylated p65)	
MAPK Pathway [13,18,20,21]	The MAPK signaling pathway family, which is involved in the promotion of cellular death, chondrocyte differentiation, matrix metalloproteinase (MMP) creation, and the formation of pro-inflammatory components.	
	ERK1/2 (Extracellular Signal-Regulated Kinase 1/2)	Kinases in the MAPK pathway that are involved in cell development and differentiation. Phosphorylated form of ERK1/2 indicates pathway activation.
	p-ERK1/2 (Phosphorylated ERK1/2	
	JNK (c-Jun N-terminal Kinase)	A member of the MAPK family involved in stress responses. Phosphorylated form of JNK indicates pathway activation.
	p-JNK (Phosphorylated JNK)	
	p38	Another MAPK member involved in stress and inflammation. Phosphorylated form of p38 indicates pathway activation.
	p-p38 (Phosphorylated p38)	
Jak-STAT Pathway [22,23]	JAK/STAT pathway is a intracellular signaling pathways, regulating cell proliferation, differentiation, and apoptosis.	

	STAT1 (Signal Transducer and Activator of Transcription 1)	Involved in a variety of cellular processes, including inflammation, cytokine response. phosphorylated STATs indicates pathway activation.
	STAT3 (Signal Transducer and Activator of Transcription 3)	
	p-STAT1 (Phosphorylated STAT1)	
	p-STAT3 (Phosphorylated STAT3)	
PI3K-Akt Pathway [20,24]	PI3K-Akt Pathway regulates various cellular processes, including cell survival, growth, proliferation, and metabolism.	
	Akt (Protein Kinase B)	The active form of Akt is phosphorylated Akt, which is involved in a variety of cellular functions.
	p-Akt (Phosphorylated Akt)	
HIF Pathway [2,25]	The HIF (Hypoxia-Inducible Factor) pathway is a vital cellular signaling cascade that plays a central role in the cellular response to low oxygen levels, or hypoxia.	
	HIF-1α (Hypoxia-Inducible Factor 1-alpha)	A key regulator of the cellular response to low oxygen levels (hypoxia).
	p-HIF-1α (Phosphorylated HIF-1α)	

References

1. Ossendorff, R.; Grad, S.; Stoddart, M.J.; Alini, M.; Schmal, H.; Südkamp, N.; Salzmann, G.M. Autologous Chondrocyte Implantation in Osteoarthritic Surroundings: TNFα and Its Inhibition by Adalimumab in a Knee-Specific Bioreactor. *Am. J. Sports Med.* **2018**, *46*, 431–440, doi:10.1177/0363546517737497.
2. Tilwani, R.K.; Vessillier, S.; Pingguan-Murphy, B.; Lee, D.A.; Bader, D.L.; Chowdhury, T.T. Oxygen tension modulates the effects of TNFα in compressed chondrocytes. *Inflamm. Res.* **2017**, *66*, 49–58, doi:10.1007/s00011-016-0991-5.
3. Bevill, S.L.; Boyer, K.A.; Andriacchi, T.P. The regional sensitivity of chondrocyte gene expression to coactive mechanical load and exogenous TNF-α stimuli. *Journal of biomechanical engineering* **2014**, *136*, 91005, doi:10.1115/1.4027937.
4. Stevens, A.L.; Wishnok, J.S.; Chai, D.H.; Grodzinsky, A.J.; Tannenbaum, S.R. A sodium dodecyl sulfate-polyacrylamide gel electrophoresis-liquid chromatography tandem mass spectrometry analysis of bovine cartilage tissue response to mechanical compression injury and the inflammatory cytokines tumor necrosis factor alpha and interleukin-1beta. *Arthritis Rheum.* **2008**, *58*, 489–500, doi:10.1002/art.23120.
5. Sui, Y.; Lee, J.H.; DiMicco, M.A.; Vanderploeg, E.J.; Blake, S.M.; Hung, H.-H.; Plaas, A.H.K.; James, I.E.; Song, X.-Y.; Lark, M.W.; et al. Mechanical injury potentiates proteoglycan catabolism induced by interleukin-6 with soluble interleukin-6 receptor and tumor necrosis factor alpha in immature bovine and adult human articular cartilage. *Arthritis Rheum.* **2009**, *60*, 2985–2996, doi:10.1002/art.24857.
6. Djouad, F.; Rackwitz, L.; Song, Y.; Janjanin, S.; Tuan, R.S. ERK1/2 activation induced by inflammatory cytokines compromises effective host tissue integration of engineered cartilage. *Tissue engineering. Part A* **2009**, *15*, 2825–2835, doi:10.1089/ten.TEA.2008.0663.
7. Yik, J.H.N.; Hu, Z.; Kumari, R.; Christiansen, B.A.; Haudenschild, D.R. Cyclin-dependent kinase 9 inhibition protects cartilage from the catabolic effects of proinflammatory cytokines. *Arthritis & rheumatology (Hoboken, N.J.)* **2014**, *66*, 1537–1546, doi:10.1002/art.38378.
8. Roman-Blas, J.A.; Stokes, D.G.; Jimenez, S.A. Modulation of TGF-beta signaling by proinflammatory cytokines in articular chondrocytes. *Osteoarthritis Cartilage* **2007**, *15*, 1367–1377, doi:10.1016/j.joca.2007.04.011.
9. Little, C.B.; Flannery, C.R.; Hughes, C.E.; Goodship, A.; Caterson, B. Cytokine induced metalloproteinase expression and activity does not correlate with focal susceptibility of articular cartilage to degeneration. *Osteoarthritis Cartilage* **2005**, *13*, 162–170, doi:10.1016/j.joca.2004.10.014.
10. Little, C.B.; Flannery, C.R.; Hughes, C.E.; Mort, J.S.; Roughley, P.J.; Dent, C.; Caterson, B. Aggrecanase versus matrix metalloproteinases in the catabolism of the interglobular domain of aggrecan in vitro. *The Biochemical journal* **1999**, *344 Pt 1*, 61–68.
11. Kim, H.-A.; Yeo, Y.; Jung, H.A.; Jung, Y.O.; Park, S.J.; Kim, S.J. Phase 2 enzyme inducer sulphoraphane blocks prostaglandin and nitric oxide synthesis in human articular chondrocytes and inhibits cartilage matrix degradation. *Rheumatology (Oxford, England)* **2012**, *51*, 1006–1016, doi:10.1093/rheumatology/ker525.
12. Morris, K.J.; Cs-Szabo, G.; Cole, A.A. Characterization of TIMP-3 in human articular talar cartilage. *Connect. Tissue Res.* **2010**, *51*, 478–490, doi:10.3109/03008201003686958.
13. Boileau, C.; Amiable, N.; Martel-Pelletier, J.; Fahmi, H.; Duval, N.; Pelletier, J.-P. Activation of proteinase-activated receptor 2 in human osteoarthritic cartilage upregulates catabolic and proinflammatory pathways capable of inducing cartilage degradation: a basic science study. *Arthritis research & therapy* **2007**, *9*, R121, doi:10.1186/ar2329.

14. Tardif, G.; Pelletier, J.-P.; Hum, D.; Boileau, C.; Duval, N.; Martel-Pelletier, J. Differential regulation of the bone morphogenic protein antagonist chordin in human normal and osteoarthritic chondrocytes. *Annals of the rheumatic diseases* **2006**, *65*, 261–264, doi:10.1136/ard.2005.037523.
15. Malemud, C.J.; Sun, Y.; Pearlman, E.; Ginley, N.M.; Awadallah, A.; Wisler, B.A.; Dennis, J.E. Monosodium Urate and Tumor Necrosis Factor-α Increase Apoptosis in Human Chondrocyte Cultures. *Rheumatology (Sunnyvale)* **2012**, *2*, 113, doi:10.4172/2161-1149.1000113.
16. Mohanraj, B.; Huang, A.H.; Yeger-McKeever, M.J.; Schmidt, M.J.; Dodge, G.R.; Mauck, R.L. Chondrocyte and mesenchymal stem cell derived engineered cartilage exhibits differential sensitivity to pro-inflammatory cytokines. *Journal of Orthopaedic Research®* **2018**, *36*, 2901–2910, doi:10.1002/jor.24061.
17. Chen, C.; Xie, J.; Rajappa, R.; Deng, L.; Fredberg, J.; Yang, L. Interleukin-1β and tumor necrosis factor-α increase stiffness and impair contractile function of articular chondrocytes. *Acta Biochim. Biophys. Sin. (Shanghai)* **2015**, *47*, 121–129, doi:10.1093/abbs/gmu116.
18. Saklatvala, J. Inflammatory signaling in cartilage: MAPK and NF-kappaB pathways in chondrocytes and the use of inhibitors for research into pathogenesis and therapy of osteoarthritis. *Curr. Drug Targets* **2007**, *8*, 305–313, doi:10.2174/138945007779940115.
19. Choi, M.-C.; Jo, J.; Park, J.; Kang, H.K.; Park, Y. NF-κB Signaling Pathways in Osteoarthritic Cartilage Destruction. *Cells* **2019**, *8*, 734, doi:10.3390/cells8070734.
20. Shakibaei, M.; John, T.; Schulze-Tanzil, G.; Lehmann, I.; Mobasheri, A. Suppression of NF-kappaB activation by curcumin leads to inhibition of expression of cyclo-oxygenase-2 and matrix metalloproteinase-9 in human articular chondrocytes: Implications for the treatment of osteoarthritis. *Biochemical pharmacology* **2007**, *73*, 1434–1445, doi:10.1016/j.bcp.2007.01.005.
21. Li, Z.; Dai, A.; Yang, M.; Chen, S.; Deng, Z.; Li, L. p38MAPK Signaling Pathway in Osteoarthritis: Pathological and Therapeutic Aspects. *J. Inflamm. Res.* **2022**, *15*, 723–734, doi:10.2147/JIR.S348491.
22. Zhou, Q.; Ren, Q.; Jiao, L.; Huang, J.; Yi, J.; Chen, J.; Lai, J.; Ji, G.; Zheng, T. The potential roles of JAK/STAT signaling in the progression of osteoarthritis. *Front. Endocrinol. (Lausanne)* **2022**, *13*, 1069057, doi:10.3389/fendo.2022.1069057.
23. Cao, Y.; Zhang, X.; Shang, W.; Xu, J.; Wang, X.; Hu, X.; Ao, Y.; Cheng, H. Proinflammatory Cytokines Stimulate Mitochondrial Superoxide Flashes in Articular Chondrocytes In Vitro and In Situ. *PloS one* **2013**, *8*, e66444, doi:10.1371/journal.pone.0066444.
24. Sun, K.; Luo, J.; Guo, J.; Yao, X.; Jing, X.; Guo, F. The PI3K/AKT/mTOR signaling pathway in osteoarthritis: a narrative review. *Osteoarthritis and Cartilage* **2020**, *28*, 400–409, doi:10.1016/j.joca.2020.02.027.
25. Fu, L.; Zhang, L.; Zhang, X.; Chen, L.; Cai, Q.; Yang, X. Roles of oxygen level and hypoxia-inducible factor signaling pathway in cartilage, bone and osteochondral tissue engineering. *Biomed. Mater.* **2021**, *16*, 22006, doi:10.1088/1748-605X/abdb73.
